# Application of the Queuing Analytic Theory in Emergency Department

**DOI:** 10.5812/atr.10526

**Published:** 2014-04-20

**Authors:** Nabil Tachfouti

**Affiliations:** 1 Laboratory of Epidemiology, Clinical Research and Community Health, Faculty of Medicine and Pharmacy, University Sidi Mohammed Ben Abdallah, Fez, Morocco

**Keywords:** Emergency Department, Queuing Theory Analysis

Dear Editor,

I read with great interest the article by Alavi-Moghaddam et al. (1) in which the authors concluded that application of the queuing theory analysis can improve movement and reduce the waiting times of patients in bottlenecks within the emergency department (ED) in Iran. As an epidemiologist in Morocco, I divide the frustration of the authors that throughput of patients in the emergency department can often be hindered by several medical and organizational factors. In Morocco, unlike most countries, emergency departments do not provide care for patients with acute severe conditions alone. Inappropriate uses of ED and underutilization of regulation centers during triage represent a burden on health system and increase the demand for ED (2, 3). I believe most emergency physicians in Morocco would presume that lack of such legal mandate concerning regulation use and appropriate consultation in ED would result in less crowd and difficulties in patient flow in emergency departments outside of Morocco.

As this article showed, work is also underway to address overcrowding in EDs and to improve patient flow. Timely access to an emergency provider is a critical dimension of quality for ED. Similar to Morocco, the Iranian emergency department analyzed in this article faces obstacles regarding personnel, lab processing and results, and critical care bed availability. We illustrate how data analysis using the queuing model, by providing a more rigorous and scientiﬁc basis for predicting patient delays to be visited by a provider, is an appropriate simulation method to analyze the way to improve ED staffing and procedures. The usefulness of queuing models in guiding ED provider scheduling decisions was demonstrated elsewhere (4, 5).

It would be interesting to study the impact of implementing solutions to the identified challenges for reducing emergency department waiting and patient holding times. Such added data would be of great interest to policy makers to maximize the effectiveness of ED and provide the best possible care to patients not only in Iranian emergency departments, but also in emergency departments worldwide.
